# Supplementary material for: Juvenile Membranous Nephropathy Developed after Human Papillomavirus (HPV) Vaccination
Source: Vaccines (Basel). 2022 Sep 1;10(9):1442. doi: 10.3390/vaccines10091442 (PMC9502366; doi:10.3390/vaccines10091442)
Supplement: Supplementary file 1 [file vaccines-10-01442-s001.zip › vaccines-1872040-supplementary.pdf]

Probability Legend:
- over 95%
- 80% to 94%
- 50% to 79%
- 20% to 49%
- 0% to 19%

Bio View:
564 Proteins in 399 Clusters
With 7618 Hidden

#	Visible?	Starred?	Bio View	Accession Number	Alternate ID	Molecular Weight	Protein Grouping Ambiguity	Fold Change by Category	Con: 1h	Dokyou Med: HPV-1-1	Dokyou Med: HPV1-2	Dokyou Med: HPV1-3	Dokyou Med: HPV2
	✓	★	Cluster of Keratin, type II cytoskeletal 6A OS=Homo sapiens OX=9606 GN=KRT6A PE=1 SV=3 (P02538)	...	KRT6A	60 kDa	★	4.9	236	2587	2468	2592	3120
	✓	★	Cluster of Keratin, type I cytoskeletal 16 OS=Homo sapiens OX=9606 GN=KRT16 PE=1 SV=4 (P08779)	...	KRT16	51 kDa	★	6.9	161	2560	2435	2526	2776
	✓	★	Keratin, type II cytoskeletal 1 OS=Homo sapiens OX=9606 GN=KRT1 PE=1 SV=6	...	KRT1	66 kDa	★	2.2	403	2078	1946	2049	2205
	✓	★	Keratin, type I cytoskeletal 9 OS=Homo sapiens OX=9606 GN=KRT9 PE=1 SV=3	...	KRT9	62 kDa	★	1.9	311	1395	1269	1324	1416
	✓	★	Cluster of Keratin, type I cytoskeletal 10 OS=Homo sapiens OX=9606 GN=KRT10 PE=1 SV=6 (P13645)	...	KRT10	59 kDa	★	1.8	133	536	509	544	609
	✓	★	Keratin, type II cytoskeletal 4 OS=Homo sapiens OX=9606 GN=KRT4 PE=1 SV=5	...	KRT4	56 kDa	★	1.8	65	277	267	275	240
	✓	★	Keratin, type II cytoskeletal 1b OS=Homo sapiens OX=9606 GN=KRT77 PE=1 SV=3	...	KRT77	62 kDa	★	2.7	36	236	233	201	239
	✓	★	Desmoplakin OS=Homo sapiens OX=9606 GN=DSP PE=1 SV=3	...	DSP	332 kDa	★	100	1	198	224	201	331
	✓	★	Keratin, type I cytoskeletal 24 OS=Homo sapiens OX=9606 GN=KRT24 PE=1 SV=1	...	KRT24	55 kDa	★	2.9	26	173	169	182	176
	✓	★	Keratin, type II cytoskeletal 78 OS=Homo sapiens OX=9606 GN=KRT78 PE=1 SV=2	...	KRT78	57 kDa	★	5.1	12	154	146	143	109
	✓	★	Junction plakoglobin OS=Homo sapiens OX=9606 GN=JUP PE=1 SV=3	...	JUP	82 kDa	★	INF		93	95	97	121
	✓	★	Cluster of Keratin, type II cytoskeletal 73 OS=Homo sapiens OX=9606 GN=KRT73 PE=1 SV=1 (Q86Y46)	...	KRT73	59 kDa	★	4.4	8	89	80	90	62
	✓	★	Complement C3 OS=Homo sapiens OX=9606 GN=C3 PE=1 SV=2	...	C3	187 kDa		9.7	3	48	55	64	108
	✓	★	Hornerin OS=Homo sapiens OX=9606 GN=HRNR PE=1 SV=2	...	HRNR	282 kDa		INF		43	53	55	101
	✓	★	Desmoglein-1 OS=Homo sapiens OX=9606 GN=DSG1 PE=1 SV=2	...	DSG1	114 kDa		INF	0	48	49	43	70
	✓	★	Fatty acid-binding protein 5 OS=Homo sapiens OX=9606 GN=FABP5 PE=1 SV=3	...	FABP5	15 kDa		INF		42	43	39	45
	✓	★	Calmodulin-like protein 5 OS=Homo sapiens OX=9606 GN=CALML5 PE=1 SV=2	...	CALML5	16 kDa		INF		34	35	38	44
	✓	★	Cluster of Complement C4-A OS=Homo sapiens OX=9606 GN=C4A PE=1 SV=2 (P0C0L4)	...	C4A	193 kDa	★	3.6	4	24	34	28	48
	✓	★	Galectin-7 OS=Homo sapiens OX=9606 GN=LGALS7 PE=1 SV=2	...	LGALS7	15 kDa		INF		20	18	22	34
	✓	★	Plakophilin-1 OS=Homo sapiens OX=9606 GN=PKP1 PE=1 SV=2	...	PKP1	83 kDa		9.5	1	15	18	21	36
	✓	★	Cystatin-A OS=Homo sapiens OX=9606 GN=CSTA PE=1 SV=1	...	CSTA	11 kDa		INF		17	22	18	25
	✓	★	Serpin B12 OS=Homo sapiens OX=9606 GN=SERPINB12 PE=1 SV=1	...	SERPIN...	46 kDa		INF		15	19	12	27
	✓	★	Protein-glutamine gamma-glutamyltransferase E OS=Homo sapiens OX=9606 GN=TGM3 PE=1 SV=4	...	TGM3	77 kDa		INF		12	14	9	24
	✓	★	Cluster of Immunoglobulin heavy constant gamma 1 OS=Homo sapiens OX=9606 GN=IGHG1 PE=1 SV=1 (P01857)	...	IGHG1	36 kDa	★	1.3	5	15	14	10	21
	✓	★	Immunoglobulin kappa constant OS=Homo sapiens OX=9606 GN=IGKC PE=1 SV=2	...	IGKC	12 kDa		2.1	2	7	10	10	13
	✓	★	Filaggrin-2 OS=Homo sapiens OX=9606 GN=FLG2 PE=1 SV=1	...	FLG2	248 kDa		4.9	1	10	12	6	18
	✓	★	Arginase-1 OS=Homo sapiens OX=9606 GN=ARG1 PE=1 SV=2	...	ARG1	35 kDa		INF		7	9	9	15
	✓	★	Complement component C9 OS=Homo sapiens OX=9606 GN=C9 PE=1 SV=2	...	C9	63 kDa		INF		8	7	8	9
	✓	★	Filaggrin OS=Homo sapiens OX=9606 GN=FLG PE=1 SV=3	...	FLG	435 kDa		INF	0	4	5	3	23

Probability Legend:
- over 95%
- 80% to 94%
- 50% to 79%
- 20% to 49%
- 0% to 19%

Bio View:
564 Proteins in 399 Clusters
With 7618 Hidden

#	Visible?	Starred?	Bio View	Accession Number	Alternate ID	Molecular Weight	Protein Grouping Ambiguity	Fold Change by Category	Con 1h	Dokyou Med HPV-1-1	Dokyou Med HPV1-2	Dokyou Med HPV1-3	Dokyou Med HPV2
	✔	★	Cluster of Complement factor H OS=Homo sapiens OX=9606 GN=CFH PE=1 SV=4 (P08603)	...	CFH	139 kDa	★	INF		3	4	2	3
	✔	★	Secretory phospholipase A2 receptor OS=Homo sapiens OX=9606 GN=PLA2R1 PE=1 SV=2	...	PLA2R1	169 kDa		INF			0		5
	✔	★	Cluster of Vimentin OS=Homo sapiens OX=9606 GN=VIM PE=1 SV=4 (P08670)	...	VIM	54 kDa	★	0.2	624	238	243	229	261
	✔	★	Cluster of Actin, cytoplasmic 2 OS=Homo sapiens OX=9606 GN=ACTG1 PE=1 SV=1 (P63261)	...	ACTG1	42 kDa	★	0.2	494	249	230	234	227
	✔	★	Cluster of Alpha-actinin-4 OS=Homo sapiens OX=9606 GN=ACTN4 PE=1 SV=2 (O43707)	...	ACTN4	105 kDa	★	0.2	264	85	92	81	153
	✔	★	Myosin-9 OS=Homo sapiens OX=9606 GN=MYH9 PE=1 SV=4	...	MYH9	227 kDa	★	0.2	213	78	85	93	185
	✔	★	Cluster of Hemoglobin subunit alpha OS=Homo sapiens OX=9606 GN=HBA1 PE=1 SV=2 (P69905)	...	HBA1	15 kDa	★	0.7	65	93	94	108	94
	✔	★	Cluster of Hemoglobin subunit beta OS=Homo sapiens OX=9606 GN=HBB PE=1 SV=2 (P68871)	...	HBB	16 kDa	★	0.5	68	73	73	76	100
	✔	★	Collagen alpha-3(VI) chain OS=Homo sapiens OX=9606 GN=COL6A3 PE=1 SV=5	...	COL6A3	344 kDa		0.2	141	45	44	35	92
	✔	★	Histone H4 OS=Homo sapiens OX=9606 GN=H4C1 PE=1 SV=2	...	H4C1	11 kDa		0.09	187	35	37	31	52
	✔	★	Cluster of Histone H2A type 1-B/E OS=Homo sapiens OX=9606 GN=H2AC4 PE=1 SV=2 (P04908)	...	H2AC4	14 kDa	★	0.1	135	25	23	22	70
	✔	★	Cluster of Tubulin beta chain OS=Homo sapiens OX=9606 GN=TUBB PE=1 SV=2 (P07437)	...	TUBB	50 kDa	★	0.2	103	36	36	34	59
	✔	★	Cluster of Histone H2B type F-S OS=Homo sapiens OX=9606 GN=H2BS1 PE=1 SV=2 (P57053)	...	H2BS1	14 kDa	★	0.2	99	46	40	46	60
	✔	★	Laminin subunit beta-2 OS=Homo sapiens OX=9606 GN=LAMB2 PE=1 SV=2	...	LAMB2	196 kDa		0.2	100	27	32	28	55
	✔	★	Cluster of Tubulin alpha-1C chain OS=Homo sapiens OX=9606 GN=TUBA1C PE=1 SV=1 (Q9BQE3)	...	TUBA1C	50 kDa	★	0.1	101	24	24	27	53
	✔	★	Prelamin-A/C OS=Homo sapiens OX=9606 GN=LMNA PE=1 SV=1	...	LMNA	74 kDa	★	0.2	92	28	31	31	50
	✔	★	Laminin subunit alpha-5 OS=Homo sapiens OX=9606 GN=LAMA5 PE=1 SV=8	...	LAMA5	400 kDa		0.2	77	25	30	31	54
	✔	★	PDZ and LIM domain protein 2 OS=Homo sapiens OX=9606 GN=PDLIM2 PE=1 SV=1	...	PDLIM2	37 kDa		0.1	80	20	23	18	28
	✔	★	Talin-1 OS=Homo sapiens OX=9606 GN=TLN1 PE=1 SV=3	...	TLN1	270 kDa	★	0.2	62	19	19	23	62
	✔	★	Cluster of Annexin A2 OS=Homo sapiens OX=9606 GN=ANXA2 PE=1 SV=2 (P07355)	...	ANXA2	39 kDa	★	0.2	61	22	25	23	38
	✔	★	Nestin OS=Homo sapiens OX=9606 GN=NES PE=1 SV=2	...	NES	177 kDa		0.2	54	24	22	27	42
	✔	★	Vinculin OS=Homo sapiens OX=9606 GN=VCL PE=1 SV=4	...	VCL	124 kDa		0.2	51	13	13	15	40
	✔	☆	Keratin, type II cytoskeletal 72 OS=Homo sapiens OX=9606 GN=KRT72 PE=1 SV=2	...	KRT72	56 kDa	★	10	6	161	150	159	98
	✔	☆	Keratin, type II cytoskeletal 80 OS=Homo sapiens OX=9606 GN=KRT80 PE=1 SV=2	...	KRT80	51 kDa	★	0.8	41	63	64	69	100
	✔	☆	Albumin OS=Homo sapiens OX=9606 GN=ALB PE=1 SV=2	...	ALB	69 kDa		0.3	74	52	56	48	71
	✔	☆	Cluster of Keratin, type II cuticular Hb5 OS=Homo sapiens OX=9606 GN=KRT85 PE=1 SV=1 (P78386)	...	KRT85	56 kDa	★	1.5	15	57	59	50	43
	✔	☆	Cluster of Glyceraldehyde-3-phosphate dehydrogenase OS=Homo sapiens OX=9606 GN=GAPDH PE=1 SV=3 (P044...	...	GAPDH	36 kDa	★	0.4	38	37	36	29	42
	✔	☆	Cluster of Ubiquitin-40S ribosomal protein S27a OS=Homo sapiens OX=9606 GN=RPS27A PE=1 SV=2 (P62979)	...	RPS27A	18 kDa	★	0.5	27	28	41	25	36
	✔	☆	Cluster of Alpha-enolase OS=Homo sapiens OX=9606 GN=ENO1 PE=1 SV=2 (P06733)	...	ENO1	47 kDa	★	0.9	12	21	21	20	37

Probability Legend:
- over 95%
- 80% to 94%
- 50% to 79%
- 20% to 49%
- 0% to 19%

Bio View:
564 Proteins in 399 Clusters
With 7618 Hidden

#	Visible?	Starred?	Bio View	Accession Number	Alternate ID	Molecular Weight	Protein Grouping Ambiguity	Fold Change by Category	Con 1h	Dokyou Med HPV-1-1	Dokyou Med HPV1-2	Dokyou Med HPV1-3	Dokyou Med HPV2
	✓	☆	Nidogen-1 OS=Homo sapiens OX=9606 GN=NID1 PE=1 SV=3	...	NID1	136 kDa		0.3	30	17	16	19	25
	✓	☆	Filamin-A OS=Homo sapiens OX=9606 GN=FLNA PE=1 SV=4	...	FLNA	281 kDa	★	0.2	39	14	16	16	32
	✓	☆	Basement membrane-specific heparan sulfate proteoglycan core protein OS=Homo sapiens OX=9606 GN=HSPG2 PE...	...	HSPG2	469 kDa		0.3	26	13	14	11	46
	✓	☆	Cluster of Heat shock 70 kDa protein 1A OS=Homo sapiens OX=9606 GN=HSPA1A PE=1 SV=1 (P0DMV8)	...	HSPA1A	70 kDa	★	0.2	34	9	16	11	25
	✓	☆	Heat shock protein beta-1 OS=Homo sapiens OX=9606 GN=HSPB1 PE=1 SV=2	...	HSPB1	23 kDa		0.5	16	12	17	16	31
	✓	☆	Agrin OS=Homo sapiens OX=9606 GN=AGRN PE=1 SV=6	...	AGRN	217 kDa		0.2	30	12	18	16	21
	✓	☆	Keratin, type II cuticular Hb2 OS=Homo sapiens OX=9606 GN=KRT82 PE=1 SV=3	...	KRT82	57 kDa	★	INF		13	19	13	37
	✓	☆	Laminin subunit gamma-1 OS=Homo sapiens OX=9606 GN=LAMC1 PE=1 SV=3	...	LAMC1	178 kDa		0.4	19	16	16	13	26
	✓	☆	Collagen alpha-1(VI) chain OS=Homo sapiens OX=9606 GN=COL6A1 PE=1 SV=3	...	COL6A1	109 kDa		0.2	26	13	10	10	24
	✓	☆	Cluster of 14-3-3 protein zeta/delta OS=Homo sapiens OX=9606 GN=YWHAZ PE=1 SV=1 (P63104)	...	YWHAZ	28 kDa	★	0.3	22	9	15	10	31
	✓	☆	Cluster of Heat shock cognate 71 kDa protein OS=Homo sapiens OX=9606 GN=HSPA8 PE=1 SV=1 (P11142)	...	HSPA8	71 kDa	★	0.5	17	12	17	15	29
	✓	☆	Fibronectin OS=Homo sapiens OX=9606 GN=FN1 PE=1 SV=5	...	FN1	272 kDa		0.5	15	12	21	11	25
	✓	☆	Cluster of O14950	...	MYL12B	20 kDa	★	0.2	25	9	11	11	18
	✓	☆	Myosin-10 OS=Homo sapiens OX=9606 GN=MYH10 PE=1 SV=3	...	MYH10	229 kDa	★	0.4	21	15	17	18	22
	✓	☆	Protein S100-A6 OS=Homo sapiens OX=9606 GN=S100A6 PE=1 SV=1	...	S100A6	10 kDa		0.3	25	14	13	16	15
	✓	☆	Myosin light polypeptide 6 OS=Homo sapiens OX=9606 GN=MYL6 PE=1 SV=2	...	MYL6	17 kDa	★	0.3	21	11	11	13	18
	✓	☆	Collagen alpha-2(IV) chain OS=Homo sapiens OX=9606 GN=COL4A2 PE=1 SV=4	...	COL4A2	168 kDa	★	0.2	31	9	9	8	24
	✓	☆	Cluster of Histone H3.1t OS=Homo sapiens OX=9606 GN=H3-4 PE=1 SV=3 (Q16695)	...	H3-4	16 kDa	★	0.1	38	6	6	6	22
	✓	☆	Dermcidin OS=Homo sapiens OX=9606 GN=DCD PE=1 SV=2	...	DCD	11 kDa		3.0	2	14	14	11	17
	✓	☆	Annexin A1 OS=Homo sapiens OX=9606 GN=ANXA1 PE=1 SV=2	...	ANXA1	39 kDa		0.4	11	8	13	10	15
	✓	☆	ATP synthase subunit beta, mitochondrial OS=Homo sapiens OX=9606 GN=ATP5F1B PE=1 SV=3	...	ATP5F1B	57 kDa		0.3	17	8	7	11	16
	✓	☆	Cluster of Peroxiredoxin-1 OS=Homo sapiens OX=9606 GN=PRDX1 PE=1 SV=1 (Q06830)	...	PRDX1	22 kDa	★	0.5	12	13	13	8	21
	✓	☆	Putative tubulin-like protein alpha-4B OS=Homo sapiens OX=9606 GN=TUBA4B PE=5 SV=2	...	TUBA4B	28 kDa	★	0.1	24	6	6	5	14
	✓	☆	Neuroblast differentiation-associated protein AHNAK OS=Homo sapiens OX=9606 GN=AHNAK PE=1 SV=2	...	AHNAK	629 kDa		0.8	11	18	20	18	31
	✓	☆	Cluster of Histone H1.2 OS=Homo sapiens OX=9606 GN=H1-2 PE=1 SV=2 (P16403)	...	H1-2	21 kDa	★	0.2	23	6	4	9	15
	✓	☆	Cluster of Tropomyosin alpha-4 chain OS=Homo sapiens OX=9606 GN=TPM4 PE=1 SV=3 (P67936)	...	TPM4	29 kDa	★	0.2	20	9	8	6	15
	✓	☆	Cluster of Putative elongation factor 1-alpha-like 3 OS=Homo sapiens OX=9606 GN=EEF1A1P5 PE=5 SV=1 (Q5VTE...	...	EEF1A1...	50 kDa	★	0.2	19	8	6	10	10
	✓	☆	Myosin-14 OS=Homo sapiens OX=9606 GN=MYH14 PE=1 SV=2	...	MYH14	228 kDa	★	0.2	19	5	6	6	25
	✓	☆	Pyruvate kinase PKM OS=Homo sapiens OX=9606 GN=PKM PE=1 SV=4	...	PKM	58 kDa	★	1.3	4	9	10	12	17

Probability Legend:

- over 95%
- 80% to 94%
- 50% to 79%
- 20% to 49%
- 0% to 19%

Bio View:
564 Proteins in 399 Clusters
With 7618 Hidden

#	Visible?	Starred?	Bio View	Accession Number	Alternate ID	Molecular Weight	Protein Grouping Ambiguity	Fold Change by Category	Con 1h	Dokyou Med HPV-1-1	Dokyou Med HPV1-2	Dokyou Med HPV1-3	Dokyou Med HPV2
	✓	☆	Peroxiredoxin-2 OS=Homo sapiens OX=9606 GN=PRDX2 PE=1 SV=5	...	PRDX2	22 kDa	★	0.4	9	10	8	4	16
	✓	☆	Gelsolin OS=Homo sapiens OX=9606 GN=GSN PE=1 SV=1	...	GSN	86 kDa		0.3	12	6	7	6	19
	✓	☆	Spectrin alpha chain, non-erythrocytic 1 OS=Homo sapiens OX=9606 GN=SPTAN1 PE=1 SV=3	...	SPTAN1	285 kDa		0.3	16	7	7	12	23
	✓	☆	Cluster of Heat shock protein HSP 90-beta OS=Homo sapiens OX=9606 GN=HSP90AB1 PE=1 SV=4 (P08238)	...	HSP90A...	83 kDa	★	0.4	12	10	14	5	12
	✓	☆	Cluster of Serpin B3 OS=Homo sapiens OX=9606 GN=SERPINB3 PE=1 SV=2 (P29508)	...	SERPINB3	45 kDa	★	INF		10	8	8	21
	✓	☆	Cluster of Moesin OS=Homo sapiens OX=9606 GN=MSN PE=1 SV=3 (P26038)	...	MSN	68 kDa	★	0.2	20	7	6	8	16
	✓	☆	Calmodulin-like protein 3 OS=Homo sapiens OX=9606 GN=CALML3 PE=1 SV=2	...	CALML3	17 kDa	★	1.2	4	10	14	9	12
	✓	☆	Plectin OS=Homo sapiens OX=9606 GN=PLEC PE=1 SV=3	...	PLEC	532 kDa	★	0.4	18	7	19	7	30
	✓	☆	Titin OS=Homo sapiens OX=9606 GN=TTN PE=1 SV=4	...	TTN	3816 kDa	★	1.8	11	50	63	32	40
	✓	☆	Annexin A5 OS=Homo sapiens OX=9606 GN=ANXA5 PE=1 SV=2	...	ANXA5	36 kDa		0.2	15	5	6	7	9
	✓	☆	ATP synthase subunit alpha, mitochondrial OS=Homo sapiens OX=9606 GN=ATP5F1A PE=1 SV=1	...	ATP5F1A	60 kDa		0.2	14	7	3	6	11
	✓	☆	Na(+)/H(+) exchange regulatory cofactor NHE-RF2 OS=Homo sapiens OX=9606 GN=SLC9A3R2 PE=1 SV=2	...	SLC9A3...	37 kDa		0.1	16	4	4	5	8
	✓	☆	Synaptopodin OS=Homo sapiens OX=9606 GN=SYNPO PE=1 SV=2	...	SYNPO	99 kDa		0.2	16	4	6	7	9
	✓	☆	Calmodulin OS=Homo sapiens GN=CALM1 PE=1 SV=2	...		?	★	0.4	9	7	11	8	11
	✓	☆	Collagen alpha-1(XVIII) chain OS=Homo sapiens OX=9606 GN=COL18A1 PE=1 SV=5	...	COL18...	178 kDa	★	0.3	13	8	6	6	12
	✓	☆	Collagen alpha-2(VI) chain OS=Homo sapiens OX=9606 GN=COL6A2 PE=1 SV=4	...	COL6A2	109 kDa		0.3	10	8	8	3	12
	✓	☆	Clathrin heavy chain 1 OS=Homo sapiens OX=9606 GN=CLTC PE=1 SV=5	...	CLTC	192 kDa	★	0.9	4	10	8	3	15
	✓	☆	Putative protein FAM90A2P OS=Homo sapiens OX=9606 GN=FAM90A2P PE=5 SV=2	...	FAM90...	50 kDa	★	INF			32	1	
	✓	☆	Peptidyl-prolyl cis-trans isomerase A OS=Homo sapiens OX=9606 GN=PPIA PE=1 SV=2	...	PPIA	18 kDa		0.3	8	4	4	3	16
	✓	☆	Transgelin-2 OS=Homo sapiens OX=9606 GN=TAGLN2 PE=1 SV=3	...	TAGLN2	22 kDa	★	0.1	15	3	2	4	12
	✓	☆	Peroxiredoxin-6 OS=Homo sapiens OX=9606 GN=PRDX6 PE=1 SV=3	...	PRDX6	25 kDa		0.4	7	4	7	4	10
	✓	☆	Cluster of Phosphoglycerate kinase 1 OS=Homo sapiens OX=9606 GN=PGK1 PE=1 SV=3 (P00558)	...	PGK1	45 kDa	★	0.3	9	4	7	7	10
	✓	☆	Collagen alpha-3(IV) chain OS=Homo sapiens OX=9606 GN=COL4A3 PE=1 SV=3	...	COL4A3	162 kDa		0.2	13	6	7	2	7
	✓	☆	78 kDa glucose-regulated protein OS=Homo sapiens GN=HSPA5 PE=1 SV=2	...		?	★	0.5	5	4	7	4	10
	✓	☆	Podocin OS=Homo sapiens OX=9606 GN=NPHS2 PE=1 SV=1	...	NPHS2	42 kDa		0.1	14	7	2	3	7
	✓	☆	Collagen alpha-1(IV) chain OS=Homo sapiens OX=9606 GN=COL4A1 PE=1 SV=4	...	COL4A1	161 kDa	★	0.6	7	9	13	10	8
	✓	☆	Collagen alpha-4(IV) chain OS=Homo sapiens OX=9606 GN=COL4A4 PE=1 SV=3	...	COL4A4	164 kDa	★	3.7	1		10	12	13
	✓	☆	Podocalyxin OS=Homo sapiens OX=9606 GN=PODXL PE=1 SV=2	...	PODXL	59 kDa		0.2	9	5	4	4	8
	✓	☆	Ezrin OS=Homo sapiens OX=9606 GN=EZR PE=1 SV=4	...	EZR	69 kDa	★	0.2	10	6	3	5	9

Probability Legend:
- over 95%
- 80% to 94%
- 50% to 79%
- 20% to 49%
- 0% to 19%

Bio View:
564 Proteins in 399 Clusters
With 7618 Hidden

#	Visible?	Starred?	Bio View	Accession Number	Alternate ID	Molecular Weight	Protein Grouping Ambiguity	Fold Change by Category	Con 1h	Dokyou Med HPV-1-1	Dokyou Med HPV1-2	Dokyou Med HPV1-3	Dokyou Med HPV2
	✓	☆	Unconventional myosin-Ic OS=Homo sapiens OX=9606 GN=MYO1C PE=1 SV=4	...	MYO1C	122 kDa	★	0.2	9	1	3	4	13
	✓	☆	Profilin-1 OS=Homo sapiens OX=9606 GN=PFN1 PE=1 SV=2	...	PFN1	15 kDa		0.3	6	2	3	4	7
	✓	☆	Protein-glutamine gamma-glutamyltransferase K OS=Homo sapiens OX=9606 GN=TGM1 PE=1 SV=4	...	TGM1	90 kDa		INF		6	6	5	13
	✓	☆	Spectrin beta chain, non-erythrocytic 1 OS=Homo sapiens OX=9606 GN=SPTBN1 PE=1 SV=2	...	SPTBN1	275 kDa	★	0.06	18	1	2		9
	✓	☆	Neprilysin OS=Homo sapiens OX=9606 GN=MME PE=1 SV=2	...	MME	86 kDa		0.02	24	0	0		5
	✓	☆	Tight junction protein ZO-1 OS=Homo sapiens OX=9606 GN=TJP1 PE=1 SV=3	...	TJP1	195 kDa	★	0.1	15	1	4	1	10
	✓	☆	Glutathione S-transferase P OS=Homo sapiens OX=9606 GN=GSTP1 PE=1 SV=2	...	GSTP1	23 kDa		0.3	5	4	4	4	3
	✓	☆	Heterogeneous nuclear ribonucleoproteins A2/B1 OS=Homo sapiens OX=9606 GN=HNRNPA2B1 PE=1 SV=2	...	HNRNP...	37 kDa	★	0.2	8	3	4	2	10
	✓	☆	Cluster of Ras-related protein Rap-1A OS=Homo sapiens OX=9606 GN=RAP1A PE=1 SV=1 (P62834)	...	RAP1A	21 kDa	★	0.3	5	2	2	3	6
	✓	☆	Alpha-2-macroglobulin OS=Homo sapiens OX=9606 GN=A2M PE=1 SV=3	...	A2M	163 kDa	★	INF	0	4	5	3	12
	✓	☆	Thioredoxin OS=Homo sapiens OX=9606 GN=TXN PE=1 SV=3	...	TXN	12 kDa		INF		4	3	5	7
	✓	☆	Cysteine and glycine-rich protein 1 OS=Homo sapiens OX=9606 GN=CSRP1 PE=1 SV=3	...	CSRP1	21 kDa		0.2	7	3	3	3	4
	✓	☆	Cluster of F-actin-capping protein subunit alpha-2 OS=Homo sapiens OX=9606 GN=CAPZA2 PE=1 SV=3 (P47755)	...	CAPZA2	33 kDa	★	0.3	5	2	3	3	7
	✓	☆	Heterogeneous nuclear ribonucleoprotein K OS=Homo sapiens OX=9606 GN=HNRNPK PE=1 SV=1	...	HNRNPK	51 kDa		0.6	3	3	6	1	7
	✓	☆	Protein S100-A9 OS=Homo sapiens OX=9606 GN=S100A9 PE=1 SV=1	...	S100A9	13 kDa		0.4	4	3	4	3	5
	✓	☆	Cluster of Calpain small subunit 1 OS=Homo sapiens OX=9606 GN=CAPNS1 PE=1 SV=1 (P04632)	...	CAPNS1	28 kDa	★	0.4	4	4	2		10
	✓	☆	Keratinocyte proline-rich protein OS=Homo sapiens OX=9606 GN=KPRP PE=1 SV=1	...	KPRP	64 kDa		INF		7	8	3	7
	✓	☆	Clusterin OS=Homo sapiens OX=9606 GN=CLU PE=1 SV=1	...	CLU	52 kDa		0.5	3	2	3	4	6
	✓	☆	Cluster of Heterogeneous nuclear ribonucleoproteins C1/C2 OS=Homo sapiens OX=9606 GN=HNRNPC PE=1 SV=4 (...	...	HNRNPC	34 kDa	★	0.3	6	2	3	3	7
	✓	☆	Endoplasmin OS=Homo sapiens OX=9606 GN=HSP90B1 PE=1 SV=1	...	HSP90B1	92 kDa	★	0.4	5	2	4	4	7
	✓	☆	Cluster of ADP-ribosylation factor 3 OS=Homo sapiens OX=9606 GN=ARF3 PE=1 SV=2 (P61204)	...	ARF3	21 kDa	★	0.2	7	1	2	3	4
	✓	☆	Annexin A6 OS=Homo sapiens OX=9606 GN=ANXA6 PE=1 SV=3	...	ANXA6	76 kDa		1.1	2	4	6	2	8
	✓	☆	Triosephosphate isomerase OS=Homo sapiens OX=9606 GN=TPI1 PE=1 SV=4	...	TPI1	27 kDa		0.9	2	1	4	3	9
	✓	☆	Protein S100-A11 OS=Homo sapiens OX=9606 GN=S100A11 PE=1 SV=2	...	S100A11	12 kDa		0.5	3	2	3	4	5
	✓	☆	Basal cell adhesion molecule OS=Homo sapiens OX=9606 GN=BCAM PE=1 SV=2	...	BCAM	67 kDa		0.2	5	2	2	1	5
	✓	☆	Filamin-B OS=Homo sapiens OX=9606 GN=FLNB PE=1 SV=2	...	FLNB	278 kDa	★	0.4	6	3	6	6	9
	✓	☆	Elongation factor 2 OS=Homo sapiens OX=9606 GN=EEF2 PE=1 SV=4	...	EEF2	95 kDa	★	1.9	1	3	3	3	10
	✓	☆	Spectrin beta chain, non-erythrocytic 4 OS=Homo sapiens OX=9606 GN=SPTBN4 PE=1 SV=2	...	SPTBN4	289 kDa	★	1.0	3	6	8	4	10
	✓	☆	Cluster of Dihydropyrimidinase-related protein 2 OS=Homo sapiens OX=9606 GN=DPYSL2 PE=1 SV=1 (Q16555)	...	DPYSL2	62 kDa	★	0.2	9	0	4	4	6

Probability Legend:
- over 95%
- 80% to 94%
- 50% to 79%
- 20% to 49%
- 0% to 19%

#	Visible?	Starred?	Bio View: 564 Proteins in 399 Clusters With 7618 Hidden	Accession Number	Alternate ID	Molecular Weight	Protein Grouping Ambiguity	Fold Change by Category	Con 1h	Dokyou Med HPV-1-1	Dokyou Med HPV1-2	Dokyou Med HPV1-3	Dokyou Med HPV2
	✓	☆	Fructose-bisphosphate aldolase A OS=Homo sapiens OX=9606 GN=ALDOA PE=1 SV=2	...	ALDOA	39 kDa	*	0.2	7	3	4	2	2
	✓	☆	Fibroblast growth factor 1 OS=Homo sapiens OX=9606 GN=FGF1 PE=1 SV=1	...	FGF1	17 kDa		0.6	2	2	2	2	6
	✓	☆	E3 ubiquitin-protein ligase HECTD1 OS=Homo sapiens OX=9606 GN=HECTD1 PE=1 SV=3	...	HECTD1	289 kDa		1.4	2	3	4	12	6
	✓	☆	Dynein axonemal heavy chain 1 OS=Homo sapiens OX=9606 GN=DNAH1 PE=2 SV=5	...	DNAH1	487 kDa	*	0.9	5	15	11	8	7
	✓	☆	Integrin alpha-3 OS=Homo sapiens OX=9606 GN=ITGA3 PE=1 SV=5	...	ITGA3	117 kDa		0.7	3	2	4	9	5
	✓	☆	Cohesin subunit SA-3 OS=Homo sapiens OX=9606 GN=STAG3 PE=1 SV=2	...	STAG3	139 kDa		0.2	8	5	4	2	2
	✓	☆	Protein bassoon OS=Homo sapiens OX=9606 GN=BSN PE=1 SV=4	...	BSN	416 kDa	*	0.9	3	13	3	3	6
	✓	☆	Ras GTPase-activating-like protein IQGAP2 OS=Homo sapiens OX=9606 GN=IQGAP2 PE=1 SV=4	...	IQGAP2	181 kDa		0.2	6	3	1	2	7
	✓	☆	Caldesmon OS=Homo sapiens OX=9606 GN=CALD1 PE=1 SV=3	...	CALD1	93 kDa		0.08	10	2		1	5
	✓	☆	Cluster of Cofilin-1 OS=Homo sapiens OX=9606 GN=CFL1 PE=1 SV=3 (P23528)	...	CFL1	19 kDa	*	0.4	3	2	3	3	2
	✓	☆	L-lactate dehydrogenase B chain OS=Homo sapiens OX=9606 GN=LDHB PE=1 SV=2	...	LDHB	37 kDa		0.6	2	1	1	5	5
	✓	☆	Integrin beta-1 OS=Homo sapiens OX=9606 GN=ITGB1 PE=1 SV=2	...	ITGB1	88 kDa		0.2	6	1	2	1	6
	✓	☆	EH domain-containing protein 3 OS=Homo sapiens OX=9606 GN=EHD3 PE=1 SV=2	...	EHD3	61 kDa	*	0.2	6	3	2	1	6
	✓	☆	Cluster of Myosin light chain 1/3, skeletal muscle isoform OS=Homo sapiens OX=9606 GN=MYL1 PE=1 SV=3 (P05...	...	MYL1	21 kDa	*	0.2	6	3	3	3	2
	✓	☆	Complement factor H-related protein 5 OS=Homo sapiens OX=9606 GN=CFHR5 PE=1 SV=1	...	CFHR5	64 kDa		INF		2	4	4	5
	✓	☆	Epiplakin OS=Homo sapiens OX=9606 GN=EPPK1 PE=1 SV=3	...	EPPK1	556 kDa	*	3.1	1	3	8	7	11
	✓	☆	Caspase recruitment domain-containing protein 11 OS=Homo sapiens OX=9606 GN=CARD11 PE=1 SV=3	...	CARD11	133 kDa		INF		3	8	3	2
	✓	☆	Cluster of Ras-related protein R-Ras OS=Homo sapiens OX=9606 GN=RRAS PE=1 SV=1 (P10301)	...	RRAS	23 kDa	*	0.2	5	1	2	2	2
	✓	☆	Filamin-C OS=Homo sapiens OX=9606 GN=FLNC PE=1 SV=3	...	FLNC	291 kDa	*	0.7	4	8	5	8	5
	✓	☆	Serotransferrin OS=Homo sapiens OX=9606 GN=TF PE=1 SV=3	...	TF	77 kDa		1.7	1	3	4	3	6
	✓	☆	Cathepsin D OS=Homo sapiens OX=9606 GN=CTSD PE=1 SV=1	...	CTSD	45 kDa		INF		2	3	1	5
	✓	☆	L-lactate dehydrogenase A chain OS=Homo sapiens OX=9606 GN=LDHA PE=1 SV=2	...	LDHA	37 kDa		0.07	7		1	1	3
	✓	☆	Microtubule-associated protein 9 OS=Homo sapiens OX=9606 GN=MAP9 PE=1 SV=3	...	MAP9	74 kDa		INF		3	9	5	10
	✓	☆	Plastin-3 OS=Homo sapiens OX=9606 GN=PLS3 PE=1 SV=4	...	PLS3	71 kDa	*	0.8	2	2	4	3	6
	✓	☆	F-box only protein 50 OS=Homo sapiens OX=9606 GN=NCCRP1 PE=1 SV=1	...	NCCRP1	31 kDa		INF		2	1	3	6
	✓	☆	Cluster of Actin-related protein 3 OS=Homo sapiens OX=9606 GN=ACTR3 PE=1 SV=3 (P61158)	...	ACTR3	47 kDa	*	0.07	8		1		5
	✓	☆	Cluster of Rab GDP dissociation inhibitor beta OS=Homo sapiens OX=9606 GN=GDI2 PE=1 SV=2 (P50395)	...	GDI2	51 kDa	*	0.3	4	3	1	4	5
	✓	☆	Septin-2 OS=Homo sapiens OX=9606 GN=SEPTIN2 PE=1 SV=1	...	SEPTIN2	41 kDa		0.3	3	2	3	1	3
	✓	☆	Cytosolic non-specific dipeptidase OS=Homo sapiens OX=9606 GN=CNDP2 PE=1 SV=2	...	CNDP2	53 kDa		0.4	3		3	3	5

Probability Legend:

- over 95%
- 80% to 94%
- 50% to 79%
- 20% to 49%
- 0% to 19%

#	Visible?	Starred?	Bio View: 564 Proteins in 399 Clusters With 7618 Hidden	Accession Number	Alternate ID	Molecular Weight	Protein Grouping Ambiguity	Fold Change by Category	Con 1h	Dokyou Med HPV-1-1	Dokyou Med HPV1-2	Dokyou Med HPV1-3	Dokyou Med HPV2
	✓	☆	Q08554	...	DSC1	100 kDa		INF		3	2	1	10
	✓	☆	Talin-2 OS=Homo sapiens OX=9606 GN=TLN2 PE=1 SV=4	...	TLN2	272 kDa	★	0.4	5	3	1		15
	✓	☆	Catalase OS=Homo sapiens OX=9606 GN=CAT PE=1 SV=3	...	CAT	60 kDa		INF	0	2	4		6
	✓	☆	Serpin A12 OS=Homo sapiens OX=9606 GN=SERPINA12 PE=1 SV=1	...	SERPIN...	47 kDa		0.1	16	4	10	2	3
	✓	☆	Transmembrane protein KIAA1109 OS=Homo sapiens OX=9606 GN=KIAA1109 PE=1 SV=2	...	KIAA11...	555 kDa		1.1	3	3	20	3	4
	✓	☆	Chloride intracellular channel protein 1 OS=Homo sapiens OX=9606 GN=CLIC1 PE=1 SV=4	...	CLIC1	27 kDa		1.1	1	3	3		4
	✓	☆	Cytoplasmic dynein 1 heavy chain 1 OS=Homo sapiens OX=9606 GN=DYNC1H1 PE=1 SV=5	...	DYNC1...	532 kDa		1.2	2	1	5	0	18
	✓	☆	Fibrinogen alpha chain OS=Homo sapiens OX=9606 GN=FGA PE=1 SV=2	...	FGA	95 kDa		INF		1	2	3	7
	✓	☆	Integrin-linked protein kinase OS=Homo sapiens OX=9606 GN=ILK PE=1 SV=2	...	ILK	51 kDa		0.2	4	2	1	1	2
	✓	☆	Tubulin polymerization-promoting protein family member 3 OS=Homo sapiens OX=9606 GN=TPPP3 PE=1 SV=1	...	TPPP3	19 kDa		0.5	2	2	2	2	3
	✓	☆	Actin-related protein 2/3 complex subunit 4 OS=Homo sapiens OX=9606 GN=ARPC4 PE=1 SV=3	...	ARPC4	20 kDa		0.2	4	1	2	3	3
	✓	☆	Prolow-density lipoprotein receptor-related protein 1 OS=Homo sapiens OX=9606 GN=LRP1 PE=1 SV=2	...	LRP1	505 kDa		0.5	4	5	3	6	5
	✓	☆	Cluster of Core histone macro-H2A.1 OS=Homo sapiens OX=9606 GN=MACROH2A1 PE=1 SV=4 (O75367)	...	MACRO...	40 kDa	★	0.3	3	1	2	1	4
	✓	☆	Ras-related protein Rab-7a OS=Homo sapiens OX=9606 GN=RAB7A PE=1 SV=1	...	RAB7A	23 kDa		INF		2	2	1	3
	✓	☆	Ubiquitin-like modifier-activating enzyme 1 OS=Homo sapiens OX=9606 GN=UBA1 PE=1 SV=3	...	UBA1	118 kDa		0.1	6	1		4	3
	✓	☆	Protein disulfide-isomerase A3 OS=Homo sapiens OX=9606 GN=PDIA3 PE=1 SV=4	...	PDIA3	57 kDa		0.1	6	1		3	3
	✓	☆	Nesprin-1 OS=Homo sapiens OX=9606 GN=SYNE1 PE=1 SV=4	...	SYNE1	1011 kDa	★	0.8	6	17	7	9	14
	✓	☆	Dynein axonemal heavy chain 9 OS=Homo sapiens OX=9606 GN=DNAH9 PE=1 SV=3	...	DNAH9	512 kDa	★	2.9	1	9	6	5	7
	✓	☆	Nesprin-2 OS=Homo sapiens OX=9606 GN=SYNE2 PE=1 SV=3	...	SYNE2	796 kDa	★	1.9	1	1	3	3	12
	✓	☆	Cluster of ADP/ATP translocase 2 OS=Homo sapiens OX=9606 GN=SLC25A5 PE=1 SV=7 (P05141)	...	SLC25A5	33 kDa	★	INF	0	4	1	3	5
	✓	☆	Phosphatidylethanolamine-binding protein 1 OS=Homo sapiens OX=9606 GN=PEBP1 PE=1 SV=3	...	PEBP1	21 kDa		1.0	1	2	3	1	3
	✓	☆	Serine/threonine-protein kinase Nek10 OS=Homo sapiens OX=9606 GN=NEK10 PE=1 SV=3	...	NEK10	133 kDa		0.2	8	3	4	2	3
	✓	☆	Reticulon-4 OS=Homo sapiens OX=9606 GN=RTN4 PE=1 SV=2	...	RTN4	130 kDa		0.3	3	5	1		3
	✓	☆	Apolipoprotein A-I OS=Homo sapiens OX=9606 GN=APOA1 PE=1 SV=1	...	APOA1	31 kDa		INF		1	2	1	5
	✓	☆	Cluster of Guanine nucleotide-binding protein G(s) subunit alpha isoforms XLas OS=Homo sapiens OX=9606 GN=...	...	GNAS	111 kDa	★	0.8	2	1	2	2	12
	✓	☆	Dynein axonemal heavy chain 2 OS=Homo sapiens OX=9606 GN=DNAH2 PE=1 SV=3	...	DNAH2	508 kDa		0.2	16	8	4	1	11
	✓	☆	Histone H1.0 OS=Homo sapiens OX=9606 GN=H1-0 PE=1 SV=3	...	H1-0	21 kDa		1.0	1	3	2	1	3
	✓	☆	Transcription factor HIVEP2 OS=Homo sapiens OX=9606 GN=HIVEP2 PE=1 SV=2	...	HIVEP2	269 kDa	★	2.5	1	5	9	5	3
	✓	☆	Septin-7 OS=Homo sapiens OX=9606 GN=SEPTIN7 PE=1 SV=2	...	SEPTIN7	51 kDa	★	0.7	1	2	1	0	4

Probability Legend:
- over 95%
- 80% to 94%
- 50% to 79%
- 20% to 49%
- 0% to 19%

Bio View:
564 Proteins in 399 Clusters
With 7618 Hidden

#	Visible?	Starred?	Bio View	Accession Number	Alternate ID	Molecular Weight	Protein Grouping Ambiguity	Fold Change by Category	Con 1h	Dokyou Med HPV-1-1	Dokyou Med HPV1-2	Dokyou Med HPV1-3	Dokyou Med HPV2
	✓	☆	Histone-lysine N-methyltransferase, H3 lysine-79 specific OS=Homo sapiens OX=9606 GN=DOT1L PE=1 SV=3	...	DOT1L	165 kDa		INF			1		12
	✓	☆	Microtubule-actin cross-linking factor 1, isoforms 1/2/3/5 OS=Homo sapiens OX=9606 GN=MACF1 PE=1 SV=4	...	MACF1	838 kDa	★	0.6	6	4	9	4	15
	✓	☆	Polypyrimidine tract-binding protein 1 OS=Homo sapiens OX=9606 GN=PTBP1 PE=1 SV=1	...	PTBP1	57 kDa	★	0.4	2	2	1		5
	✓	☆	Histone-lysine N-methyltransferase 2D OS=Homo sapiens OX=9606 GN=KMT2D PE=1 SV=2	...	KMT2D	593 kDa	★	1.0	2	1	1	6	12
	✓	☆	Heat shock protein 75 kDa, mitochondrial OS=Homo sapiens OX=9606 GN=TRAP1 PE=1 SV=3	...	TRAP1	80 kDa		1.2	1	2		5	4
	✓	☆	Cluster of Septin-11 OS=Homo sapiens OX=9606 GN=SEPTIN11 PE=1 SV=3 (Q9NVA2)	...	SEPTIN11	49 kDa	★	0.3	2		3	3	0
	✓	☆	ADP-ribosylation factor 6 OS=Homo sapiens OX=9606 GN=ARF6 PE=1 SV=2	...	ARF6	20 kDa		0.7	1	1	3		3
	✓	☆	Annexin A4 OS=Homo sapiens OX=9606 GN=ANXA4 PE=1 SV=4	...	ANXA4	36 kDa		INF	0	1			5
	✓	☆	Cluster of Phosphatidylinositol-binding clathrin assembly protein OS=Homo sapiens OX=9606 GN=PICALM PE=1 S...	...	PICALM	71 kDa	★	0.8	1	2	1	2	3
	✓	☆	Treacle protein OS=Homo sapiens OX=9606 GN=TCOF1 PE=1 SV=3	...	TCOF1	152 kDa		INF	0	6	2	4	2
	✓	☆	Heterogeneous nuclear ribonucleoprotein U OS=Homo sapiens OX=9606 GN=HNRNPU PE=1 SV=6	...	HNRNPU	91 kDa		0.3	2	2	3		1
	✓	☆	LIM and SH3 domain protein 1 OS=Homo sapiens OX=9606 GN=LASP1 PE=1 SV=2	...	LASP1	30 kDa		0.6	1		1	2	3
	✓	☆	Centromere-associated protein E OS=Homo sapiens OX=9606 GN=CENPE PE=1 SV=2	...	CENPE	316 kDa	★	0.7	2	5	4	0	5
	✓	☆	Cluster of Trypsin-1 OS=Homo sapiens OX=9606 GN=PRSS1 PE=1 SV=1 (P07477)	...	PRSS1	27 kDa	★	0.6	1		1	2	3
	✓	☆	Golgin subfamily B member 1 OS=Homo sapiens OX=9606 GN=GOLGB1 PE=1 SV=2	...	GOLGB1	376 kDa		INF	0	2	1	2	10
	✓	☆	Poly(rC)-binding protein 1 OS=Homo sapiens OX=9606 GN=PCBP1 PE=1 SV=2	...	PCBP1	37 kDa		0.1	3	1	1	1	1
	✓	☆	Kinesin-like protein KIF14 OS=Homo sapiens OX=9606 GN=KIF14 PE=1 SV=1	...	KIF14	186 kDa		0.2	3	2	3	0	1
	✓	☆	Malate dehydrogenase, cytoplasmic OS=Homo sapiens OX=9606 GN=MDH1 PE=1 SV=4	...	MDH1	36 kDa		INF	0	1	3	2	3
	✓	☆	Protocadherin Fat 1 OS=Homo sapiens OX=9606 GN=FAT1 PE=1 SV=2	...	FAT1	506 kDa		0.3	3	1	0	2	7
	✓	☆	Nucleosome-remodeling factor subunit BPTF OS=Homo sapiens OX=9606 GN=BPTF PE=1 SV=3	...	BPTF	338 kDa		1.4	1	2	8	2	0
	✓	☆	Transitional endoplasmic reticulum ATPase OS=Homo sapiens OX=9606 GN=VCP PE=1 SV=4	...	VCP	89 kDa		INF		0	5	4	4
	✓	☆	PDZ and LIM domain protein 5 OS=Homo sapiens OX=9606 GN=PDLIM5 PE=1 SV=5	...	PDLIM5	64 kDa		0.2	2			1	4
	✓	☆	Proteasome subunit alpha type-6 OS=Homo sapiens OX=9606 GN=PSMA6 PE=1 SV=1	...	PSMA6	27 kDa		INF		1	1		3
	✓	☆	Chloride intracellular channel protein 5 OS=Homo sapiens OX=9606 GN=CLIC5 PE=1 SV=3	...	CLIC5	47 kDa	★	0.1	3		1		3
	✓	☆	Transgelin OS=Homo sapiens OX=9606 GN=TAGLN PE=1 SV=4	...	TAGLN	23 kDa		0.2	3	1			5
	✓	☆	Mucin-16 OS=Homo sapiens OX=9606 GN=MUC16 PE=1 SV=3	...	MUC16	1519 kDa		1.0	2	14	2	1	1
	✓	☆	Fermitin family homolog 2 OS=Homo sapiens OX=9606 GN=FERMT2 PE=1 SV=1	...	FERMT2	78 kDa	★	0.4	2		2	1	5
	✓	☆	Ninein-like protein OS=Homo sapiens OX=9606 GN=NINL PE=1 SV=2	...	NINL	156 kDa		INF			2	3	6
	✓	☆	Transforming growth factor beta-1-induced transcript 1 protein OS=Homo sapiens OX=9606 GN=TGFB1I1 PE=1 SV...	...	TGFB1I1	50 kDa		0.6	1		1		5

Probability Legend:
- over 95%
- 80% to 94%
- 50% to 79%
- 20% to 49%
- 0% to 19%

#	Visible?	Starred?	Bio View: 564 Proteins in 399 Clusters With 7618 Hidden	Accession Number	Alternate ID	Molecular Weight	Protein Grouping Ambiguity	Fold Change by Category	Con: 1h	Dokyou Med: HPV-1-1	Dokyou Med: HPV1-2	Dokyou Med: HPV1-3	Dokyou Med: HPV2
	✓	☆	Dolichyl-diphosphooligosaccharide--protein glycosyltransferase subunit 1 OS=Homo sapiens OX=9606 GN=RPN1	...	RPN1	69 kDa		0.2	2	0		1	4
	✓	☆	Collagen alpha-1(I) chain OS=Homo sapiens OX=9606 GN=COL1A1 PE=1 SV=5	...	COL1A1	139 kDa	★	0.4	3				12
	✓	☆	Unconventional myosin-Ib OS=Homo sapiens OX=9606 GN=MYO1B PE=1 SV=3	...	MYO1B	132 kDa		INF		0		0	10
	✓	☆	Nebulin OS=Homo sapiens OX=9606 GN=NEB PE=1 SV=5	...	NEB	773 kDa		0.9	3	3	3	10	10
	✓	☆	Unconventional myosin-XV OS=Homo sapiens OX=9606 GN=MYO15A PE=1 SV=2	...	MYO15A	395 kDa		0.7	3	5	9	2	2
	✓	☆	Nebulette OS=Homo sapiens OX=9606 GN=NEBL PE=1 SV=1	...	NEBL	116 kDa		0.6	2	5		4	2
	✓	☆	Protein PRRC2C OS=Homo sapiens OX=9606 GN=PRRC2C PE=1 SV=4	...	PRRC2C	317 kDa	★	0.5	4	3	2	10	2
	✓	☆	SH3 domain-binding glutamic acid-rich-like protein 2 OS=Homo sapiens OX=9606 GN=SH3BGRL2 PE=1 SV=2	...	SH3BGR...	12 kDa		0.4	1		1	1	2
	✓	☆	Purine nucleoside phosphorylase OS=Homo sapiens OX=9606 GN=PNP PE=1 SV=2	...	PNP	32 kDa		INF		1	2		3
	✓	☆	Very long-chain specific acyl-CoA dehydrogenase, mitochondrial OS=Homo sapiens OX=9606 GN=ACADVL PE=1 S...	...	ACADVL	70 kDa		INF	0	1	1		2
	✓	☆	Nuclear receptor corepressor 2 OS=Homo sapiens OX=9606 GN=NCOR2 PE=1 SV=3	...	NCOR2	274 kDa	★	0.5	2	1	4		4
	✓	☆	Fibulin-1 OS=Homo sapiens OX=9606 GN=FBLN1 PE=1 SV=4	...	FBLN1	77 kDa		0.5	1	2	1		2
	✓	☆	Ras GTPase-activating-like protein IQGAP1 OS=Homo sapiens OX=9606 GN=IQGAP1 PE=1 SV=1	...	IQGAP1	189 kDa		0.6	1	2	0	0	4
	✓	☆	Cluster of 60S acidic ribosomal protein P0 OS=Homo sapiens OX=9606 GN=RPLP0 PE=1 SV=1 (P05388)	...	RPLP0	34 kDa	★	INF	0	1		1	3
	✓	☆	Tenascin OS=Homo sapiens OX=9606 GN=TNC PE=1 SV=3	...	TNC	241 kDa		INF			1	1	6
	✓	☆	Collagen alpha-1(VII) chain OS=Homo sapiens OX=9606 GN=COL7A1 PE=1 SV=2	...	COL7A1	295 kDa		0.3	3		0		10
	✓	☆	Alstrom syndrome protein 1 OS=Homo sapiens OX=9606 GN=ALMS1 PE=1 SV=4	...	ALMS1	461 kDa		1.7	1	11	2	1	1
	✓	☆	G protein-regulated inducer of neurite outgrowth 1 OS=Homo sapiens OX=9606 GN=GPRIN1 PE=1 SV=2	...	GPRIN1	102 kDa		1.2	1		2	7	2
	✓	☆	Extracellular matrix protein 1 OS=Homo sapiens OX=9606 GN=ECM1 PE=1 SV=2	...	ECM1	61 kDa		INF	0	1		1	4
	✓	☆	Thioredoxin-dependent peroxide reductase, mitochondrial OS=Homo sapiens OX=9606 GN=PRDX3 PE=1 SV=3	...	PRDX3	28 kDa		0.10	3		1		2
	✓	☆	40S ribosomal protein S3 OS=Homo sapiens OX=9606 GN=RPS3 PE=1 SV=2	...	RPS3	27 kDa		INF			0	1	3
	✓	☆	Bromodomain and WD repeat-containing protein 1 OS=Homo sapiens OX=9606 GN=BRWD1 PE=1 SV=4	...	BRWD1	263 kDa		0.3	2	1			6
	✓	☆	Glutamyl aminopeptidase OS=Homo sapiens OX=9606 GN=ENPEP PE=1 SV=3	...	ENPEP	109 kDa		INF			2		6
	✓	☆	Prohibitin OS=Homo sapiens OX=9606 GN=PHB PE=1 SV=1	...	PHB	30 kDa		INF				2	4
	✓	☆	Transforming growth factor-beta-induced protein ig-h3 OS=Homo sapiens OX=9606 GN=TGFBI PE=1 SV=1	...	TGFBI	75 kDa		0.1	2				3
	✓	☆	Proteasome subunit beta type-3 OS=Homo sapiens OX=9606 GN=PSMB3 PE=1 SV=2	...	PSMB3	23 kDa		INF				1	3
	✓	☆	NLR family member X1 OS=Homo sapiens OX=9606 GN=NLRX1 PE=1 SV=1	...	NLRX1	108 kDa		INF			1		3
	✓	☆	6-phosphogluconate dehydrogenase, decarboxylating OS=Homo sapiens OX=9606 GN=PGD PE=1 SV=3	...	PGD	53 kDa		0.09	2				2
	✓	☆	F-actin-capping protein subunit beta OS=Homo sapiens OX=9606 GN=CAPZB PE=1 SV=4	...	CAPZB	31 kDa		INF				1	3

Probability Legend:

- over 95%
- 80% to 94%
- 50% to 79%
- 20% to 49%
- 0% to 19%

Bio View:
564 Proteins in 399 Clusters
With 7618 Hidden

#	Visible?	Starred?	Bio View	Accession Number	Alternate ID	Molecular Weight	Protein Grouping Ambiguity	Fold Change by Category	Con 1h	Dokyou Med HPV-1-1	Dokyou Med HPV1-2	Dokyou Med HPV1-3	Dokyou Med HPV2
	✓	☆	EH domain-containing protein 4 OS=Homo sapiens OX=9606 GN=EHD4 PE=1 SV=1	...	EHD4	61 kDa	★	0.2	3	0	2		3
	✓	☆	Cluster of Eukaryotic initiation factor 4A-I OS=Homo sapiens OX=9606 GN=EIF4A1 PE=1 SV=1 (P60842)	...	EIF4A1	46 kDa	★	0.4	1				4
	✓	☆	60S ribosomal protein L6 OS=Homo sapiens OX=9606 GN=RPL6 PE=1 SV=3	...	RPL6	33 kDa		INF	0	1		0	3
	✓	☆	Nephronectin OS=Homo sapiens OX=9606 GN=NPNT PE=2 SV=3	...	NPNT	62 kDa		INF			1	0	5
	✓	☆	Transaldolase OS=Homo sapiens OX=9606 GN=TALDO1 PE=1 SV=2	...	TALDO1	38 kDa		0.5	1				5
	✓	☆	Abnormal spindle-like microcephaly-associated protein OS=Homo sapiens OX=9606 GN=ASPM PE=1 SV=2	...	ASPM	410 kDa		0.8	1	1	1	0	6
	✓	☆	Cilia- and flagella-associated protein 54 OS=Homo sapiens OX=9606 GN=CFAP54 PE=2 SV=3	...	CFAP54	352 kDa		0.4	2	0	1	2	4
	✓	☆	WD repeat and FYVE domain-containing protein 3 OS=Homo sapiens OX=9606 GN=WDFY3 PE=1 SV=2	...	WDFY3	395 kDa		INF	0	2	0	1	4
	✓	☆	Nucleoprotein TPR OS=Homo sapiens OX=9606 GN=TPR PE=1 SV=3	...	TPR	267 kDa		0.4	2	1	1	1	4
	✓	☆	Fibronectin type III domain-containing protein 1 OS=Homo sapiens OX=9606 GN=FNDC1 PE=2 SV=4	...	FNDC1	206 kDa	★	INF	0	0	2	5	1
	✓	☆	Thyroid receptor-interacting protein 11 OS=Homo sapiens OX=9606 GN=TRIP11 PE=1 SV=3	...	TRIP11	228 kDa		0.7	1	0	0	4	3
	✓	☆	Pre-mRNA-processing-splicing factor 8 OS=Homo sapiens OX=9606 GN=PRPF8 PE=1 SV=2	...	PRPF8	274 kDa		0.4	2	5	2	0	
	✓	☆	Envoplakin OS=Homo sapiens OX=9606 GN=EVPL PE=1 SV=3	...	EVPL	232 kDa	★	0.5	2	0	7		2
	✓	☆	Tenascin-X OS=Homo sapiens OX=9606 GN=TNXB PE=1 SV=5	...	TNXB	458 kDa		0.5	2			3	7
	✓	☆	Aldo-keto reductase family 1 member A1 OS=Homo sapiens OX=9606 GN=AKR1A1 PE=1 SV=3	...	AKR1A1	37 kDa		0.1	2	1			2
	✓	☆	Msx2-interacting protein OS=Homo sapiens OX=9606 GN=SPEN PE=1 SV=1	...	SPEN	402 kDa		0.3	3	1	8	0	0
	✓	☆	Rootletin OS=Homo sapiens OX=9606 GN=CROCC PE=1 SV=1	...	CROCC	229 kDa	★	INF	0	1	1	0	8
	✓	☆	Cluster of NUT family member 2E OS=Homo sapiens OX=9606 GN=NUTM2E PE=3 SV=3 (B1AL46)	...	NUTM2E	94 kDa	★	0.4	1		3	0	1
	✓	☆	E3 ubiquitin-protein ligase MYCBP2 OS=Homo sapiens OX=9606 GN=MYCBP2 PE=1 SV=4	...	MYCBP2	514 kDa		0.3	3	0	7		0
	✓	☆	Cluster of Complement receptor type 1 OS=Homo sapiens OX=9606 GN=CR1 PE=1 SV=3 (P17927)	...	CR1	224 kDa	★	0.6	1		4		2
	✓	☆	Adenylyl cyclase-associated protein 1 OS=Homo sapiens OX=9606 GN=CAP1 PE=1 SV=5	...	CAP1	52 kDa		INF		2	1		6
	✓	☆	Protein POF1B OS=Homo sapiens OX=9606 GN=POF1B PE=1 SV=3	...	POF1B	68 kDa		INF		1	4		0
	✓	☆	Hemicentin-2 OS=Homo sapiens OX=9606 GN=HMCN2 PE=2 SV=3	...	HMCN2	542 kDa		0.4	3	1	0		12
	✓	☆	Annexin A7 OS=Homo sapiens OX=9606 GN=ANXA7 PE=1 SV=3	...	ANXA7	53 kDa		INF				1	3
	✓	☆	Fructose-bisphosphate aldolase B OS=Homo sapiens OX=9606 GN=ALDOB PE=1 SV=2	...	ALDOB	39 kDa		0.2	1				2
	✓	☆	60 kDa heat shock protein, mitochondrial OS=Homo sapiens OX=9606 GN=HSPD1 PE=1 SV=2	...	HSPD1	61 kDa		0.9	1	3		0	6
	✓	☆	Proteasome subunit beta type-4 OS=Homo sapiens OX=9606 GN=PSMB4 PE=1 SV=4	...	PSMB4	29 kDa		0.3	1			1	2
	✓	☆	Cluster of Sodium/potassium-transporting ATPase subunit alpha-1 OS=Homo sapiens OX=9606 GN=ATP1A1 PE=...	...	ATP1A1	113 kDa	★	0.2	3		1		4
	✓	☆	Tropomodulin-3 OS=Homo sapiens OX=9606 GN=TMOD3 PE=1 SV=1	...	TMOD3	40 kDa		INF		0	2		2

Probability Legend:
- over 95%
- 80% to 94%
- 50% to 79%
- 20% to 49%
- 0% to 19%

Bio View:
564 Proteins in 399 Clusters
With 7618 Hidden

#	Visible?	Starred?	Bio View	Accession Number	Alternate ID	Molecular Weight	Protein Grouping Ambiguity	Fold Change by Category	Con	Dokyou Med			
									1h	HPV-1-1	HPV1-2	HPV1-3	HPV2
	✓		Hydrocephalus-inducing protein homolog OS=Homo sapiens OX=9606 GN=HYDIN PE=1 SV=3	...	HYDIN	576 kDa		INF	0	0	0		9
	✓		Heterogeneous nuclear ribonucleoprotein R OS=Homo sapiens OX=9606 GN=HNRNPR PE=1 SV=1	...	HNRNPR	71 kDa	★	0.4	1		1		3
	✓		Actin-related protein 2 OS=Homo sapiens OX=9606 GN=ACTR2 PE=1 SV=1	...	ACTR2	45 kDa		INF			1		2
	✓		Alpha-parvin OS=Homo sapiens OX=9606 GN=PARVA PE=1 SV=1	...	PARVA	42 kDa	★	INF	0			2	4
	✓		Carbonic anhydrase 2 OS=Homo sapiens OX=9606 GN=CA2 PE=1 SV=2	...	CA2	29 kDa		0.3	1				3
	✓		U4/U6.U5 tri-snRNP-associated protein 1 OS=Homo sapiens OX=9606 GN=SART1 PE=1 SV=1	...	SART1	90 kDa		INF			1	4	0
	✓		Inverted formin-2 OS=Homo sapiens OX=9606 GN=INF2 PE=1 SV=2	...	INF2	136 kDa		INF		0			6
	✓		Acetyl-CoA acetyltransferase, mitochondrial OS=Homo sapiens OX=9606 GN=ACAT1 PE=1 SV=1	...	ACAT1	45 kDa		INF	0				3
	✓		Serpin B13 OS=Homo sapiens OX=9606 GN=SERPINB13 PE=1 SV=2	...	SERPIN...	44 kDa		INF	0				3
	✓		Lysosome-associated membrane glycoprotein 1 OS=Homo sapiens OX=9606 GN=LAMP1 PE=1 SV=3	...	LAMP1	45 kDa		INF					5
	✓		Ras suppressor protein 1 OS=Homo sapiens OX=9606 GN=RSU1 PE=1 SV=3	...	RSU1	32 kDa		INF					4
	✓		DNA-dependent protein kinase catalytic subunit OS=Homo sapiens OX=9606 GN=PRKDC PE=1 SV=3	...	PRKDC	469 kDa		0.5	4	8	3	2	5
	✓		StAR-related lipid transfer protein 9 OS=Homo sapiens OX=9606 GN=STARD9 PE=1 SV=3	...	STARD9	516 kDa	★	0.8	2	2	7	1	6
	✓		Dynein axonemal heavy chain 10 OS=Homo sapiens OX=9606 GN=DNAH10 PE=1 SV=4	...	DNAH10	515 kDa	★	1.0	1	4	0	2	4
	✓		Obscurin OS=Homo sapiens OX=9606 GN=OBSCN PE=1 SV=3	...	OBSCN	868 kDa		0.8	1	1	5	0	1
	✓		Xin actin-binding repeat-containing protein 2 OS=Homo sapiens OX=9606 GN=XIRP2 PE=1 SV=2	...	XIRP2	382 kDa		0.6	1	1	1	2	2
	✓		PDZ and LIM domain protein 7 OS=Homo sapiens OX=9606 GN=PDLIM7 PE=1 SV=1	...	PDLIM7	50 kDa		INF		3	1		4
	✓		Cardiomyopathy-associated protein 5 OS=Homo sapiens OX=9606 GN=CMYA5 PE=1 SV=3	...	CMYA5	449 kDa		0.7	1	1	1	1	4
	✓		Vacuolar protein sorting-associated protein 13D OS=Homo sapiens OX=9606 GN=VPS13D PE=1 SV=2	...	VPS13D	492 kDa		INF	0	1	3		6
	✓		Protein AHNAK2 OS=Homo sapiens OX=9606 GN=AHNAK2 PE=1 SV=2	...	AHNAK2	617 kDa		INF	0	1	3		8
	✓		Serine/arginine repetitive matrix protein 2 OS=Homo sapiens OX=9606 GN=SRRM2 PE=1 SV=2	...	SRRM2	300 kDa	★	0.3	4	10	0		2
	✓		Dynein axonemal heavy chain 3 OS=Homo sapiens OX=9606 GN=DNAH3 PE=2 SV=1	...	DNAH3	471 kDa		INF		3	0	0	6
	✓		Tudor domain-containing protein 6 OS=Homo sapiens OX=9606 GN=TDRD6 PE=2 SV=2	...	TDRD6	237 kDa		INF	0	2	4		0
	✓		Ryanodine receptor 2 OS=Homo sapiens OX=9606 GN=RYR2 PE=1 SV=3	...	RYR2	565 kDa		INF	0		1	7	1
	✓		26S proteasome non-ATPase regulatory subunit 1 OS=Homo sapiens OX=9606 GN=PSMD1 PE=1 SV=2	...	PSMD1	106 kDa		INF		1			2
	✓		Apolipoprotein B-100 OS=Homo sapiens OX=9606 GN=APOB PE=1 SV=2	...	APOB	516 kDa		INF	0	9		0	0
	✓		Malate dehydrogenase, mitochondrial OS=Homo sapiens OX=9606 GN=MDH2 PE=1 SV=3	...	MDH2	36 kDa		INF	0	0	1		3
	✓		Heterogeneous nuclear ribonucleoprotein M OS=Homo sapiens OX=9606 GN=HNRNPM PE=1 SV=3	...	HNRNPM	78 kDa		0.4	1	1			3
	✓		ATP-dependent RNA helicase A OS=Homo sapiens OX=9606 GN=DHX9 PE=1 SV=4	...	DHX9	141 kDa		INF	0		1		2

Probability Legend:

- over 95%
- 80% to 94%
- 50% to 79%
- 20% to 49%
- 0% to 19%

Bio View:
564 Proteins in 399 Clusters
With 7618 Hidden

#	Visible?	Starred?	Bio View	Accession Number	Alternate ID	Molecular Weight	Protein Grouping Ambiguity	Fold Change by Category	Con: 1h	Dokyou Med: HPV-1-1	Dokyou Med: HPV1-2	Dokyou Med: HPV1-3	Dokyou Med: HPV2
	✓	☆	Protein inturned OS=Homo sapiens OX=9606 GN=INTU PE=1 SV=2	...	INTU	106 kDa		INF		0	1		3
	✓	☆	Proteasome subunit alpha type-3 OS=Homo sapiens OX=9606 GN=PSMA3 PE=1 SV=2	...	PSMA3	28 kDa		INF			1		3
	✓	☆	Na(+)/H(+) exchange regulatory cofactor NHE-RF1 OS=Homo sapiens OX=9606 GN=SLC9A3R1 PE=1 SV=4	...	SLC9A3...	39 kDa		0.0	2				0
	✓	☆	CAP-Gly domain-containing linker protein 1 OS=Homo sapiens OX=9606 GN=CLIP1 PE=1 SV=2	...	CLIP1	162 kDa	★	INF		0	7		
	✓	☆	Cluster of RNA-binding motif protein, X chromosome OS=Homo sapiens OX=9606 GN=RBMX PE=1 SV=3 (P38159)	...	RBMX	42 kDa	★	INF		1			3
	✓	☆	Collagen alpha-1(II) chain OS=Homo sapiens OX=9606 GN=COL2A1 PE=1 SV=3	...	COL2A1	142 kDa	★	INF			0		8
	✓	☆	Cluster of Serine/threonine-protein phosphatase 2A 65 kDa regulatory subunit A beta isoform OS=Homo sapiens ...	...	PPP2R1B	66 kDa	★	INF				1	2
	✓	☆	Serpin H1 OS=Homo sapiens OX=9606 GN=SERPINH1 PE=1 SV=2	...	SERPIN...	46 kDa		INF	0				2
	✓	☆	Complement C1q subcomponent subunit B OS=Homo sapiens OX=9606 GN=C1QB PE=1 SV=3	...	C1QB	27 kDa		INF					2
	✓	☆	Cell division control protein 42 homolog OS=Homo sapiens OX=9606 GN=CDC42 PE=1 SV=2	...	CDC42	21 kDa		INF					2
	✓	☆	Signal transducer and activator of transcription 1-alpha/beta OS=Homo sapiens OX=9606 GN=STAT1 PE=1 SV=2	...	STAT1	87 kDa		INF		2			
	✓	☆	Protein disulfide-isomerase A6 OS=Homo sapiens OX=9606 GN=PDIA6 PE=1 SV=1	...	PDIA6	48 kDa		INF					2
	✓	☆	Barrier-to-autointegration factor OS=Homo sapiens OX=9606 GN=BANF1 PE=1 SV=1	...	BANF1	10 kDa		INF					2
	✓	☆	Aldehyde dehydrogenase, mitochondrial OS=Homo sapiens OX=9606 GN=ALDH2 PE=1 SV=2	...	ALDH2	56 kDa	★	INF					6
	✓	☆	Proliferation marker protein Ki-67 OS=Homo sapiens OX=9606 GN=MKI67 PE=1 SV=2	...	MKI67	359 kDa		1.0	1	2	2	2	3
	✓	☆	Cluster of RANBP2-like and GRIP domain-containing protein 2 OS=Homo sapiens OX=9606 GN=RGPD2 PE=2 SV=1...	...	RGPD2	197 kDa	★	1.5	1	2	2	1	10
	✓	☆	Histone-lysine N-methyltransferase 2B OS=Homo sapiens OX=9606 GN=KMT2B PE=1 SV=1	...	KMT2B	294 kDa		0.1	2	0			3
	✓	☆	Acrosomal protein KIAA1210 OS=Homo sapiens OX=9606 GN=KIAA1210 PE=2 SV=3	...	KIAA12...	187 kDa		0.4	1	0	3		1
	✓	☆	Cullin-associated NEDD8-dissociated protein 1 OS=Homo sapiens OX=9606 GN=CAND1 PE=1 SV=2	...	CAND1	136 kDa		0.2	1	0	0		2
	✓	☆	Hexokinase-1 OS=Homo sapiens OX=9606 GN=HK1 PE=1 SV=3	...	HK1	102 kDa	★	0.3	1			1	2
	✓	☆	Trifunctional enzyme subunit alpha, mitochondrial OS=Homo sapiens OX=9606 GN=HADHA PE=1 SV=2	...	HADHA	83 kDa		INF		1		1	3
	✓	☆	Papilin OS=Homo sapiens OX=9606 GN=PAPLN PE=2 SV=4	...	PAPLN	138 kDa		INF			1		4
	✓	☆	Chromodomain-helicase-DNA-binding protein 8 OS=Homo sapiens OX=9606 GN=CHD8 PE=1 SV=5	...	CHD8	291 kDa	★	INF	0	0			5
	✓	☆	Adenosylhomocysteinase OS=Homo sapiens OX=9606 GN=AHCY PE=1 SV=4	...	AHCY	48 kDa		INF		1			2
	✓	☆	Cluster of Heterogeneous nuclear ribonucleoprotein A1-like 2 OS=Homo sapiens OX=9606 GN=HNRNPA1L2 PE=2 S...	...	HNRNP...	34 kDa	★	INF					5
	✓	☆	Microtubule-associated protein 4 OS=Homo sapiens OX=9606 GN=MAP4 PE=1 SV=3	...	MAP4	121 kDa		INF					4
	✓	☆	Cluster of Glutathione S-transferase A1 OS=Homo sapiens OX=9606 GN=GSTA1 PE=1 SV=3 (P08263)	...	GSTA1	26 kDa	★	INF					6
	✓	☆	Cluster of Coatomer subunit gamma-2 OS=Homo sapiens OX=9606 GN=COPG2 PE=1 SV=1 (Q9UBF2)	...	COPG2	98 kDa	★	INF					3
	✓	☆	T-complex protein 1 subunit zeta OS=Homo sapiens OX=9606 GN=CCT6A PE=1 SV=3	...	CCT6A	58 kDa		INF					2

Probability Legend:

- over 95%
- 80% to 94%
- 50% to 79%
- 20% to 49%
- 0% to 19%

Bio View:
564 Proteins in 399 Clusters
With 7618 Hidden

#	Visible?	Starred?	Bio View	Accession Number	Alternate ID	Molecular Weight	Protein Grouping Ambiguity	Fold Change by Category	Con 1h	Dokyou Med HPV-1-1	Dokyou Med HPV1-2	Dokyou Med HPV1-3	Dokyou Med HPV2
	☑	☆	Retinal dehydrogenase 1 OS=Homo sapiens OX=9606 GN=ALDH1A1 PE=1 SV=2	...	ALDH1...	55 kDa	★	INF					2
	☑	☆	OTU domain-containing protein 5 OS=Homo sapiens OX=9606 GN=OTUD5 PE=1 SV=1	...	OTUD5	61 kDa		INF			4		
	☑	☆	Protein piccolo OS=Homo sapiens OX=9606 GN=PCLO PE=1 SV=5	...	PCLO	561 kDa	★	0.7	1	4	0	1	1
	☑	☆	Ryanodine receptor 3 OS=Homo sapiens OX=9606 GN=RYR3 PE=1 SV=3	...	RYR3	552 kDa		0.2	2		0	1	3
	☑	☆	Protocadherin Fat 2 OS=Homo sapiens OX=9606 GN=FAT2 PE=1 SV=2	...	FAT2	479 kDa		INF	0	1	8		
